# Extracts from an Address

**Published:** 1880-11

**Authors:** H. Campian

**Affiliations:** President


### EXTRACTS FROM AN ADDRESS.

READ BEFORE A SPECIAL GENERAL MEETING OF THE MIDLAND COUNTIES BRANCH OF THE BRITISH DENTAL ASSOCIATION, HELD OCT. 6, 1880.

BY H. CAMPIAN, PRESIDENT.

The objects of the branch, you see by the by-laws, are four-fold:

1. To render assistance, as far as possible, in carrying out the provisions of the Dentists' Act.

2. The general consideration of subjects affecting the interests of the profession.

3. The reading and discussion of papers on Dental Surgery and Mechanics.

4. The cultivation of a generous professional spirit amongst practitioners throughout the district,

With respect to the first object, I think the purport of the Act is so clear that it requires but few remarks from me. It is impossible to draw any definite line where the help of an unqualified person must cease and that of the duly qualified practitioner commence. Any one who dresses a wound or a bruise, or reduces a dislocation, practises surgery, and no one could for a moment imagine that a law would ever be passed to prevent his doing so; and in like manner, we could not expect that the legislature would ever allow the drawing of a tooth or any other similar operation to be made a penal action, although the person so doing may be said to be practising Dentistry; but as soon as any one endeavors to make the public believe he is a qualified practitioner, by assuming the title of Dentist or any other name implying the possession of the Dental diploma, so soon he becomes amenable to the law; the act thus guaranteeing to the public, that *for the future*, any one who claims the professional title shall of necessity have obtained the necessary qualification. Any person, or any number of persons, who may wish to put the act in force in any particular case can do so, but before taking action it will be necessary to obtain the sanction of the Medical Council. This, at first sight may seem to be an unnecessary precaution, yet I think on further consideration you will admit that it is a very wise provision, as it entirely prevents the possibility of any one being proceeded against from personal pique or any improper motive; and if in any case it is thought desirable that a person's name should be removed from the register, all that is necessary is to collect sufficient *reliable* evidence, and transmit it to the central board in London, who will bring the matter before the Medical Council, the only body by whom such action can be taken. And in cases where these proceedings may be necessary, I am sure they can be carried out without earning for ourselves the opprobrium of acting as spies or professional police, which some have already been willing to assign to us.

With regard to the second object of the Society, we must all feel that the interests of the profession will at times require the careful consideration of its members, and necessitate the existance of some organized body which shall be able to act with the authority of the bulk of its members, and for this, no better scheme can be devised than the one we are now so much interested in—the formation of a central society, with recognized branches in the more distant parts of the country, constituting an organization by which the feeling of the majority of the profession may at any time be ascertained on any question that may arise bearing on the well being of the profession.

In the third object, "The reading and discussion of papers on Dental Surgery and Mechanics," the surgery you will notice is placed first, and justly so, as the higher branch; though in early times, and I fear even to a more recent date, the order in importance was more frequently reversed in practice. The mere mechanical calling of former times—for in its infancy Dentistry was little else—has now been developed into a profession and gained admission within the sacred portals of the College at Lincoln's Inn Fields, and it is for the present and future generations to prove by the exercise of their highest mental as well as mechanical faculties, that the profession is worthy of the position which has been accorded to it.

It has been noticed by those who most frequently attend the meetings of our specialty, that papers on mechanical subjects are more easily procured, and often prove more attractive than those on surgical subjects, but this will no doubt become less as the educational facilities of the present day are brought to bear more and more on the whole body of the profession. Surely the preservation of the natural organs is of far more importance and value to the patient than the substitution of others, however efficiently supplied.

What should we think of the surgeon who allowed himself to be deterred from directing all his energies to the preservation and restoration to health, of a diseased or injured limb, by the thought that an artificial substitute could be provided for it. No! No! Whatever our politics may be let our surgery be conservative. Far be it from me to appear to undervalue any branch of my profession, for no one can have been long in practice without having experienced the well-earned gratification derived from noticing the relief from pain, and in many instances the perfect restoration to health, that follows the substitution of efficient members in the place of useless and diseased ones; but far greater is the credit and the higher the appreciation of the patient, when the diseased natural organs themselves can be preserved and restored to a state of efficiency; and how great are the facilities for so doing in the present day, compared with the early reminiscences of many of our older brethren. All of us who were fortunate enough to hear the interesting paper read before the General Meeting of the Society in August last, must have been forcibly struck with the contrast between the paucity and quality of the instruments there mentioned, and the appliances of the present day. What would have been the feelings—I might almost say the bewilderment of the practitioner therein described, could he have been transported into one of the large depots with which we are so familiar. The numerous and beautifully adapted instruments for the variety of operations unknown in these days. The admirably adjusted forces for each form of tooth, the endless variety of excavators and pluggers, the wonderfully delicate nerve extractors, the rubber dam and its adjustments, the saliva pump, the electric mallet, the improvement in our chairs, and the greatest of all boons, both to patient and operator, the Morrison engine, the name of the inventor of which instrument ought to be indelibly inscribed in letters of gold in the Archives of Dentistry to say nothing of the application of vulcanite and celluloid, and the many ingenious appliances for the workshop. Surely these should lead us to value the benefits we enjoy, and teach us to strive to use them to the best of our ability, not influenced by the thought of self-glorification in attempting to surpass all others, in the performance of this or that brilliant operation, but ever remembering that the ultimate aim of all our efforts should be the increased amount of good which we are thereby enabled to accomplish for the benefit of our suffering fellow-creatures.

Although the *transactions* of the Odontological Society contain a very voluminous and valuable collection of papers bearing on one specialty, there still remains numerous subjects and modes of operating which may be made productive of profitable discussion. Such are the replantation of teeth now attracting so much attention, the erosion of the surfaces of the teeth of which so little is known in the present day. The various improvements in the materials for filling, and amongst a variety of subjects, far too numerous to be mentioned here, the startling announcement of the so-called New Departure Creed.

As this last subject which I think we all feel greatly interested in, and also bears on the branch of Dentistry which we are now considering I will venture a few remarks on some articles of the accepted and new departure creeds as tabulated in the *Dental Cosmos*.

I am not aware that the so-called accepted creed has been the recognized standard of practice in this country. The doctrine that gold, and nothing but gold, should be used for permanent fillings, has certainly been extensively promulgated by those of our Transatlantic brethren who have settled in this country; and I cannot but look upon this new departure as the natural reaction which might be expected to follow the over anxiety to build up large adhesive gold fillings, on fragments of weak and often disorganized teeth, totally unsuited for such an operation; but until I can see some stronger reason than has been hitherto adduced by the advocates of this new departure, I must, in any case suitable for a good gold filling, confess my unwillingness to abandon for any other of the fillings now in use, a material that we know from past experience is capable, when judiciously applied, of preserving and restoring to a state of efficiency, in some cases for a period of twenty years or even for a much longer time, teeth which otherwise would have been lost in about the same number of months. For the efficient use of this material much must of course depend on the manipulative ability of the operator, but it has often seemed to me a matter of doubt, whether, in the case of those large adhesive gold fillings, the patient has received an equivalent for the tedious and necessarily expensive operation that has been undergone.

The choice between contour fillings and separation of the teeth in the case of approximal cavities, must, I think, depend in a measure on the aptitude and judgment of the operator, since the two plans when successfully performed, may be made equally efficient. The latter however — separation — seems to afford greater facilities for operating, and also for cleanliness in those patients who are unable or unwilling to devote the time and attention necessary for that object.

The professed incompatibility of gold as a filling material with tooth bone, seems to me to be at variance with the frequent success which we all must have experienced, in the use of that filling in cavities on the labial surface of the roots of the upper incisors. I mention this particular position as being easy of access for operating, and also as being one where the enamel, tooth bone, gold and saliva, are in constant contact with each other. In those cases where the success of the operation has not equalled our expectations, in endeavoring to estimate the probable incompatibility of the filling material as a cause of failure, it is, I think, necessary to consider whether in the ordinary operation for filling, the whole of the diseased dentine has been so thoroughly removed, as to admit of its proving a satisfactory test case, for the microscope reveals to us a change in the dentine in the sides of a decayed cavity, even when it appears sound to the eye, and also the feel of the instrument; and in those portions of the margin of the cavity which we undercut for the retention of the filling, we have the dentine not only denuded of its nutrient covering, the periosteum, but also deprived of its nutrition from the pulp by the severance of the tubes of the dentine, and the intervention of the filling material, and in this deteriorated condition, in all probability still exposed to the continuous action of the same deleterious influences (whatever they may have been) which first caused the decay; and I cannot but think that the failure when it occurs, is in a greater measure due to the above named causes, rather than to the incompatibility of the gold with the tooth bone; but with all the success that has attended the use of gold for so many years, we must still, I am sure, be willing to admit the want of a perfect plastic filling, which shall be able to withstand the friction of mastication, and also the deleterious action of the fluids of the mouth.

The dictum that, "a tooth than can be so treated as to be satisfactorily filled with anything, is worth filling," is one, I think, that all who value conservative surgery must readily agree with.

That "unskilful and unscrupulous Dentists fill with tin covered with gold, thereby causing galvanic action, pulpitis, death of the pulp, abscess, and loss of the tooth," has certainly not been an accepted creed in this country, for I was early taught the use of this material both alone, and in combination with gold, when I first commenced the study of my profession with Mr. Sheffield, of Exeter, and long experience, and the retention of a tin filling in my own mouth, in perfect condition for over five-and-twenty years, has fully proved to me the value of this metal. When used in combination with gold, and exposed to contact with the fluids of the mouth, it certainly undergoes a chemical change, becoming nearly black in color, but without staining the tooth as some amalgams do; it also becomes harder, and cuts harsh like an amalgam filling, but the change does not appear to cause any alteration in its bulk, or in any respect to interfere with its efficiency as a filling, nor have I ever found it produce any of the evils suggested in the creed, and I cannot but think that it would be more used, were it not for the universal prejudice that exists in the mind of the public in favor of gold, partly arising from the magical charm which resides in the word gold, and partly from the nothing but gold theory (if I may so call it), which has been so largely spread by our American brethren.

That "a filling may be the best known for a tooth and yet leak badly," seems a simple admission that in some cases bad *is* the best we can do for them. If experience has taught us anything, it certainly has proved that it is the leak which does the mischief; and as in warfare no fortress is considered stronger than its weakest part, so it is with a tooth that is filled. A small leaking point will soon undermine the filling, and prove its ruin.

The statement that "gutta-percha *properly used* is the *most permanent* filling material which we possess," must make us wish to know the meaning of the words "properly used," for the rapidity with which it wears away, in any position in which it is subjected to friction in mastication, seems to me to prevent the possibility of its ever being permanent.

The force of the article which says that "a poor gutta percha filling, *in its proper place*, is better than a *good* gold one," seems to depend on the question as to what is the proper place for a gutta-percha filling; surely not the place where we can insert a good gold plug, if by "good," is meant one that is not only solid, but also sufficiently tight to exclude all moisture from the cavity.

It certainly is rather startling to hear from the country from which have come the severest criticisms on the use and the users of amalgam fillings, the admission that "amalgam per se, is an excellent filling material." With amalgams, as with the other plastic materials, *a perfect* filling of its kind has yet to be discovered, for a measure of uncertainty seems to exist in all of them; but as a proof that an amalgam *may* make a good useful filling, I can say that I have seen more than one apparently in a perfect condition after thirty years' wear, and I cannot but think that much of the discredit attaching to its use, arises from its being the filling we naturally have recourse to in all cases of doubts and difficulty.

That "the use of plastic filling material tends to lower the standard of Dentistry, thereby diminishing its sphere of usefulness," is scarcely a fair way of stating the matter. The indiscriminate use of them would certainly do so, but the judicious selection of them in cases unsuited for the use of gold, and not necessarily interfere with the acquirement of the manipulative ability necessary for making good gold fillings, nor is it desirable that it should do so, as the power of manipulation required for the successful use of gold, must tend to perfect the powers of the operator in the use of all plastic materials, and must thus extend the sphere of usefulness of that Dentistry which has for its standard of excellency, ability to save teeth.

Time has only permitted me briefly to notice some portions of this new creed, to show that it contains much that might be productive of profitable discussion, but I think from what has been said, we may reasonably conclude that each material has its own particular advantages, and that until we are in possession of a filling suitable for universal application, a judicious selection is necessary on the part of the operator in each particular case.

